# Fibropreventive and Antifibrotic Effects of Uncaria gambir on Rats with Pulmonary Fibrosis

**DOI:** 10.1155/2022/6721958

**Published:** 2022-06-26

**Authors:** Desdiani Desdiani, Iris Rengganis, Samsuridjal Djauzi, Agus Setiyono, Mohamad Sadikin, Sri Widia A. Jusman, Nuryati Chairani Siregar, Suradi Suradi, Putri C. Eyanoer

**Affiliations:** ^1^Faculty of Medicine, Universitas Sultan Ageng Tirtayasa, Serang, Banten, Indonesia; ^2^Department of Internal Medicine, Faculty of Medicine, Universitas Indonesia, Jakarta, Indonesia; ^3^Faculty of Veterinary Medicine, IPB University, Bogor, Indonesia; ^4^Department of Biochemistry and Molecular Biology, Faculty of Medicine, Universitas Indonesia, Jakarta, Indonesia; ^5^Department of Anatomical Pathology, Faculty of Medicine, Universitas Indonesia, Jakarta, Indonesia; ^6^Department of Pulmonology and Respiratory Medicine, Faculty of Medicine, Universitas Sebelas Maret, Surakarta, Indonesia; ^7^Department of Community Medicine, Faculty of Medicine, Universitas Sumatera Utara, Medan, Indonesia

## Abstract

Pulmonary fibrosis causes scar tissue formation that disrupts the functioning of the lungs. *Uncaria gambir* (Hunter) Roxb (hereafter gambir)—a plant native to West Sumatra in Indonesia—contains flavonoid (+)-catechin, which has strong antioxidant activity and can be used to combat pulmonary fibrosis. This random in vivo experimental study analyzed the antifibrotic effect of gambir on the lungs of rats with bleomycin-induced fibrosis. The subjects were 10 groups of 10-week-old male rats weighing around 200–250 g. All groups were terminated at the end of the seventh week or on day 50. The lungs were cleaned, and tissues were taken to analyze inflammatory cell counts and TGF-*β*1 levels using bronchoalveolar lavage (BAL) with ELISA; type I collagen and tissue inhibitor of metalloproteinase 1 (TIMP-1) levels using immunohistochemistry (IHC); and activation of NF-*κ*B using ELISA and Western blot assays. The most severe histopathological characteristic based on the modified Ashcroft score was in the bleomycin group (BG), whereas the mildest was in the 262 mg/kg of the bodyweight antifibrotic gambir-dosed group (AF G262). The results showed a significant difference in the BAL inflammatory cell count (*p*=0.017; *p* < 0.05). AF G262 differed most from the other antifibrotic groups in terms of the number of inflammatory cells (0.63), TGF-*β*1 levels (3.80), and NF-*κ*B levels (0.48), followed by the 131 mg/kg of the bodyweight antifibrotic gambir-dosed group (AF G131), which also differed most from other antifibrotic groups in terms of NF-*κ*B (0.48), TIMP-1 (11.74), and collagen I (14.50) levels. Western blot analysis showed that the fibropreventive and antifibrotic groups had a specific band size of p65, whereas no specific band binding existed in the control group. This study concluded that the administration of AF G262 could improve fibrosis by lysing the extracellular matrix (ECM) in rat lungs.

## 1. Introduction

Pulmonary fibrosis is a condition that causes scar tissue formation. This condition involves an infiltration of inflammatory cells, a proliferation of fibroblasts and reactive oxygen species (ROS), and excessive extracellular matrix accumulation in pulmonary parenchymal tissue, which may inhibit the pulmonary functioning [1]. The pathogenesis of fibrosis also implicates the growth factor TGF-*β* as an important fibrosis mediator and being the potential target in antifibrotic therapy [2]. Based on data from the Indonesian Pulmonary Doctors Association (Persatuan Dokter Paru Indonesia; PDPI), the prevalence of pulmonary fibrosis in Indonesia in 2017 reached 6.26–7.73 percent per 1,000,000 population and was predicted to increase. Only 700–1500 patients were detected due to a lack of treatment [3]. Green tea, with its catechin (flavonoid) content, proved to have fibrolytic and fibropreventive effects in a study on rats [1]. *Uncaria gambir* (Hunter) Roxb (hereafter gambir) is a member of the Rubiaceae family and is abundant in Southeast Asia, especially in the Sumatra region. This plant's flavonoid (+)-catechin is known for its strong antioxidant activity [4]. Catechin can repair alcohol-induced liver damage through the mechanism of the in vivo NF-*κ*B anti-inflammatory pathway [5]. Based on the previous study [1], gambir can also be used for pulmonary fibrosis treatment; therefore, an in vivo study using an animal model was required to examine its mechanism as an antifibrotic and/or fibropreventive substance. Intratracheal bleomycin induction is the best preclinical test to study fibrosis in animal models. Bleomycin can induce oxidative stress in pulmonary tissue, triggering an inflammatory reaction and the formation of fibrotic tissues [2]. As a new therapy for pulmonary fibrosis (which is currently irreversible), the administration of gambir was expected to prevent and treat extensive pulmonary fibrosis in rat models. Green tea was used for fibropreventive and antifibrotic comparison.

## 2. Materials and Methods

### 2.1. Ethics Statement

This study was conducted according to the requirements of the Helsinki Declaration, and approval was obtained from the Health Research Ethics Committee, Faculty of Veterinary Medicine IPB University, Bogor, Indonesia, under registration number 114/KEH/SKE/XII/2018, and the Health Research Ethics Committee, Faculty of Medicine, Universitas Indonesia.

### 2.2. Rats and Gambir Dosing

This random in vivo analytical study, with an experimental design, was performed on healthy 10-week-old Sprague–Dawley male rats weighing ±200–250 g each. Prior to treatment, the rats were acclimatized for 14 d in individual polycarbonate cages covered with wood shavings containing 1 rat per cage. The rats received standard food and drink, ad libitum, and an albendazole anthelmintic. The room temperature was kept at 25–30°C, with 80–90% humidity, under 12 h of light and 12 h of darkness. Besides ensuring adaptation to treatment conditions, acclimatization helped in selecting sick animals. The dose of bleomycin used was 4 mg/kg body weight, given intratracheally. The doses of gambir given were 65 mg, 131 mg, and 262 mg/kg body weight orally. In this study, the subjects were divided into 10 groups of 3 rats each, as follows:

1. Negative control group (NG)
2. Bleomycin group (BG); rats only given 4 mg/kg of bodyweight bleomycin from day 0 to day 21
3. Fibropreventive group (FP GT): rats given 4 mg/kg of bodyweight bleomycin on day 8 and day 28 + green tea daily for 49 days
4. Antifibrotic group (AF GT): rats given 4 mg/kg of bodyweight of bleomycin on day 0 and day 21 + green tea from day 15 to day 49
5. Fibropreventive group (FP G65): rats given 4 mg/kg of bodyweight of bleomycin on day 8 and day 28 + 65 mg/kg of bodyweight of gambir daily until day 49
6. Fibropreventive group (FP G131): rats given 4 mg/kg of bodyweight of bleomycin on day 8 and day 28 + 131 mg/kg of bodyweight of gambir daily until day 49
7. Fibropreventive group (FP G262): rats given 4 mg/kg of bodyweight of bleomycin on day 8 and day 28 + 262 mg/kg of bodyweight of gambir daily until day 49
8. Antifibrotic group (AF G65): rats given 4 mg/kg of bodyweight of bleomycin on day 0 and day 21 + 65 mg/kg of bodyweight of gambir daily from day 15 to day 49
9. Antifibrotic group (AF G131): rats given 4 mg/kg of bodyweight of bleomycin on day 0 and day 21 + 131 mg/kg of bodyweight of gambir daily from day 15 to day 49
10. Antifibrotic group (AF G262): rats given 4 mg/kg of bodyweight of bleomycin on day 0 and day 21 + 262 mg/kg of bodyweight of gambir daily from day 15 to day 49

The dose of green tea used was 125 mg/kg of bodyweight given orally. All rats were terminated at the end of the seventh week or on day 50 with injectable anesthetics methods using the ketamine lethal dose.

### 2.3. Lung Tissue Histology

Prior to staining, the organs were fixed with paraffin to maintain the cell texture and structure during the cutting and staining process. The hydration stage aimed to facilitate the entry of dyes because water-soluble materials were used. Staining to observe lung cells/tissue was performed with hematoxylin eosin using Mayer's hematoxylin reagent and eosin solution (HE), and with Masson's trichome (MT) using the Carrazzi's hematoxylin reagent, orange G 0.75%, Ponceau xylidine fuchsin, 2.5% phosphotungstic acid solution, and aniline blue. Staining was carried out on tissue section thicknesses of 3–5 mm. Thereafter, dehydration was conducted using a suitable xylene-soluble material (DPX). Tissue staining was followed by observing the collagen structure formation under a microscope and evaluating the fibrosis in each treatment group. Fibrosis images were analyzed using a modified Aschroft score.

Immunohistochemical staining was carried out using the Dako Real EnVision (Dako) procedure with a special object glass coated with poly-L-lysine. The percentage of fibrosis area was calculated, and the protein expression level was tested using immunohistochemical examination. The percentage of fibrosis area was the ratio of the total area of brown collagen to the total area of one visual field, and the calculation of extracellular collagen I and TIMP-1 expression levels was carried out using image analysis techniques with the ImageJ program.

### 2.4. Bronchoalveolar Lavage Fluid Collection, Cell Counts, and TGF-*β*1 ELISA

The lungs of all the groups were cleaned and prepared to analyze the inflammatory cells and TGF-*β*1 levels using BAL and the ELISA method. The debris was separated from the BAL fluid at a centrifugation speed of 5000 rpm for 5 min. Absorption was read with an ELISA reader at a wavelength of 450 nm. The smear was placed under a microscope, and cells were counted manually using a manual hemocytometer. The results of absorbance measurements were calculated as averages and then tabulated, prior to preparing a concentration diagram of the absorbance. After obtaining the results of the formula, using absorbance data, the sample concentration was calculated.

### 2.5. NF-*κ*B ELISA and Western Blot Analysis

Activation of NF-*κ*B was assessed using ELISA and Western blot assays. The ELISA procedure was carried out after tissue was homogenized by centrifugation for 15 min at a speed of 10,000 rpm to prepare an antibody cocktail as an ELISA reagent and standard preparation. The results were read with the ELISA reader at 450 nm. The results of absorbance measurements were calculated as averages and tabulated, and then a concentration diagram of the absorbance was prepared. After obtaining the results of the formula, using absorbance data, the sample concentration was calculated.

Lung tissue was homogenized in PBS containing a protease inhibitor. After homogenization, it was centrifuged for 15 min at 14,000 rpm at 4°C. The supernatants of lung tissue were collected, and the protein concentration of each sample was measured using a bicinchoninic acid (BCA) assay kit according to the BSA standard. Then, 150 *µ*g of protein for each sample was dissolved in 10% Tris-glycine SDS polyacrylamide gel. The protein bands were transferred to nitrocellulose membranes, and after 1 h of incubation in liquid (5% dry milk and Tris-buffered saline/Tween 20) at room temperature, membranes were incubated for 24 h with anti-TGF-*β*1. Secondary antibodies were added at a dilution of 1 : 10,000 and incubated at room temperature for 1 h. The densitometry of nitrocellulose membrane, antibodies, and secondary antibodies was conducted with ImageQuant LAS4000.

The resulting data were analyzed with SPSS v. 20. An ANOVA test was performed to check for any differences between variables, followed by posthoc tests if the results were significant.

## 3. Results

### 3.1. Formation of Fibrotic Tissue in the Lung Tissue of Mice Exposed to Bleomycin

The baseline characteristics of subjects included demographic characteristics (bodyweight and lung weight) and pulmonary macroscopic images, as shown in Table 1.

**Figure 1:**
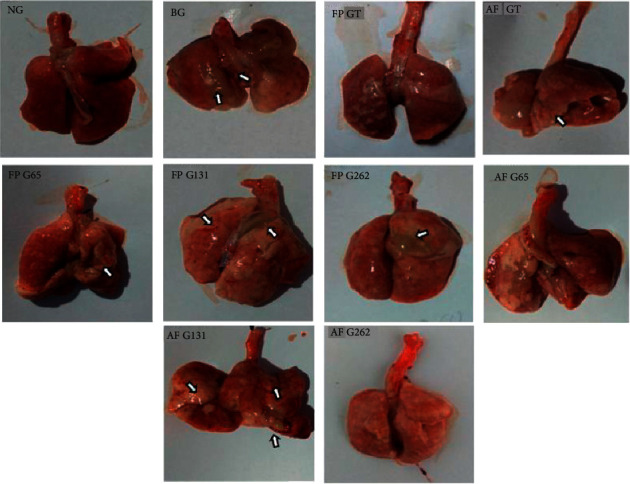
Macroscopic image of pulmonary tissue of the subjects. Arrow indicated bleeding and lumps in lung tissue. The BG group was smaller compared to NG, although had heavier relative weight (0.016 vs. 0.018). FP GT, AF GT, FP G65, AF G65, and AF G262 showed less damage compared to other groups.

The subjects were 10-week-old male rats assigned to 10 groups of 3 rats each with bodyweights of approximately 200–250 g. The lowest bodyweight mean average was in FP G262, at 210 g, whereas the highest was in FP GT, at 246 g. After termination, the lungs were weighed, and their macroscopic characteristics assessed. The lung weights of all subjects were approximately 2.8–4.1 g. Relative organ weight was calculated as the lung weight divided by the bodyweight. NG had the highest relative organ weight, at 0.018 g, whereas FP G262 and AF G262 had the lowest relative organ weights, at 0.013. The macroscopic imaging of the subjects is shown in Figure 1.

### 3.2. Effects of Gambir on Bleomycin-Induced Pulmonary Fibrosis

**Figure 2:**
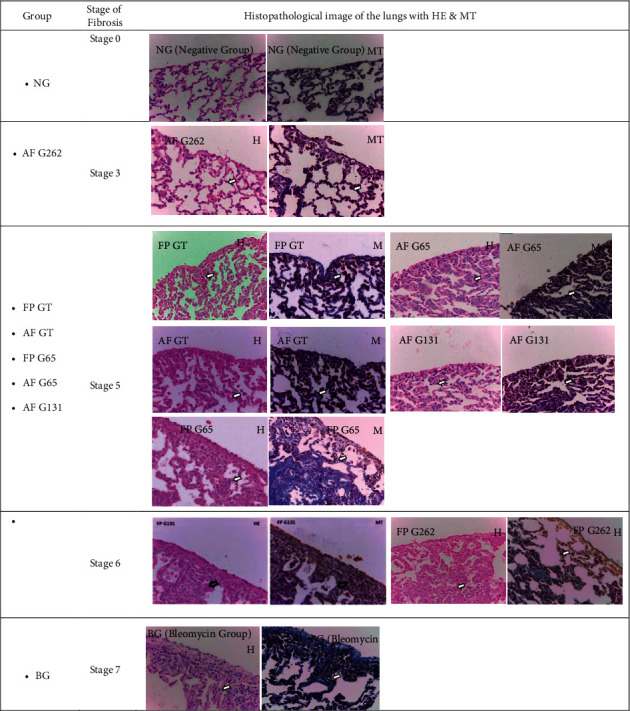
Histopathological changes in pulmonary tissues of treatment groups using hematoxylin-eosin (HE) and Masson trichome (MT) staining.

According to the histopathological characteristics, each group was assessed using a modified Ashcroft score to determine the stages of fibrosis. The most severe histopathological characteristic based on the modified Ashcroft score was observed in BG. In gambir groups, the most severe was observed in FP G131 and FP G262, as shown in Figure 2.

### 3.3. Gambir Alters Inflammatory Cell Profiles in Lungs

**Figure 3:**
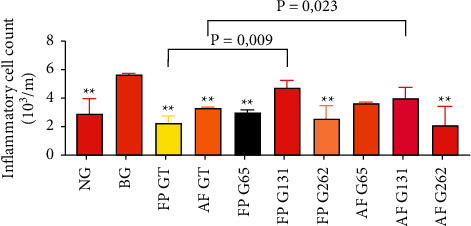
Comparison of inflammatory cell count between groups. Noted significant decrease of BAL inflammatory cell count caused by the administration of green tea and gambir compared to the bleomycin group (^*∗∗*^*p* < 0.05 vs. BG).

The inflammatory cell count results from the bronchoalveolar lavage (BAL) assessment for groups 1–10 were 2.90 (2.3–4.0), 5.70 (5.0–5.8), 2.25 (2.0–2.8), 3.35 (2.7–3.4), 3.0 (2.8–3.2), 4.75 (4.6–5.3), 2.60 (2.0–3.5), 3.70 (1.6–3.8), 4.00 (3.9–4.8), and 2.10 (2.0–3.5), respectively. The statistical test showed a significant difference in the BAL inflammatory cell count (*p*=0.017; *p* < 0.05). The posthoc analysis of the inflammatory cell count differences between the fibropreventive, antifibrotic green tea, and gambir groups revealed significant differences between several groups, as shown in Figure 3.

### 3.4. Gambir Has No Effect on Preventing TGF-*β*1 Expression and Activation

**Figure 4:**
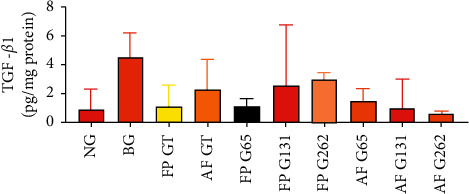
Comparison of TGF-*β*1 BAL between groups. There was insignificant difference of TGF-*β*1 BAL level between groups (*p* > 0.05).

The results of the TGF-*β*1-level measurements (pg/mg protein) for groups 1–10 were 0.88 (0.3–2.3), 4.52 (2.6–6.2), 1.14 (0.1–2.6), 2.30 (1.0–4.4), 1.12 (0.4–1.6), 2.58 (1.2–6.7), 2.97 (1.6–3.5), 1.50 (0.3–2.3), 1.02 (0.1–3.1), and 0.64 (0.5–0.8), respectively. The statistical results of all groups showed insignificant differences in TGF-*β*1 levels (*p*=0.117; *p* > 0.05), as shown in Figure 4.

**Figure 5:**
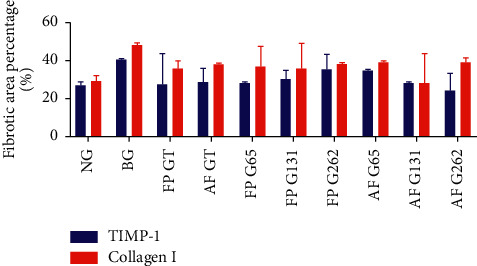
Comparison of TIMP-1 and collagen I expression between groups. There was insignificant difference in TIMP-1 and collagen I expression between groups (*p* > 0.05).

The results of the TIMP-1 expression assessment using Image *J* for groups 1–10 were 26.77 (16.76–28.77), 40.5 (32.44–40.6), 27.28 (21.36–43.34), 28.56 (22.62–35.70), 27.87 (23.14–28.41), 30.37 (20.97–34.70), 35.30 (32.46–43.13), 34.58 (26.50–35.51), 27.84 (22.28–28.20), and 24.52 (23.03–33.22), respectively. Additionally, the results of the collagen I expression assessment using Image *J* for groups 1–10 were 29.2 (26.35–32.03), 48.36 (44.51–49.02), 36.04 (26.17–39.99), 38.02 (29.40–38.64), 36.82 (34.88–47.71), 35.59 (32.53–48.90), 38.4 (29.82–38.77), 39.46 (33.43–39.93), 28.41 (26.42–43.57), and 39.43 (37.95–41.40), respectively. The statistical results of all groups revealed no significant differences in TIMP-1 and collagen I expression levels (*p*=0.31 and *p*=0.168; *p* > 0.05), as shown in Figure 5.

### 3.5. Gambir Does Not Prevent Bleomycin-Induced Activation of NF-*κ*B

**Figure 6:**
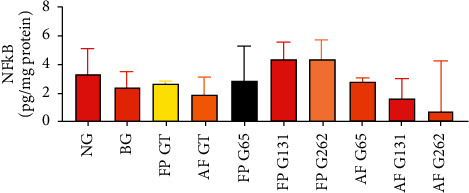
Comparison of pulmonary tissue NF-*κ*B level between groups. There was insignificant difference in pulmonary tissue NF-*κ*B level between groups (*p* > 0.05).

The results of the NF-*κ*B assessment (pg/mg protein) for groups 1–10 were 3.34 (2.5–5.1), 2.41 (1.0–3.5), 2.62 (2.2–2.8), 1.89 (1.9–3.2), 2.89 (2.2–5.3), 4.38 (3.4–5.6), 4.36 (3.3–5.7), 2.79 (1.7–3.1), 1.59 (1.0–3.0), and 0.70 (0.6–4.2), respectively. The statistical results of all groups showed no significant differences in NF-*κ*B levels (*p*=0.134; *p* > 0.05), as shown in Figure 6.

The mean discrepancy value was obtained by subtracting the mean value of the bleomycin group from the mean value of the treatment group. The largest discrepancy in the inflammatory cell count was observed in FP GT. The largest discrepancies in the TGF-*β*1 and NF-*κ*B levels were seen in AF G262. The largest discrepancies in TIMP-1 and collagen expression levels were seen in AF G131. Among the fibropreventive groups, FP GT had the largest discrepancy in terms of the inflammatory cell count (3.15), NF-*κ*B (−0.22) and collagen I expression (13.23) levels, followed by FP G65, which had the largest discrepancy among the fibropreventive groups in terms of the TGF-*β*1 (3.38) and TIMP-1 expression (11.37) levels.

Increasing or decreasing the gambir dose had no effect on reducing the inflammatory cell count. By contrast, the lower the gambir dose, the better its influence in reducing the TGF-*β*1 and collagen I expression levels (i.e., decreasing the mean discrepancies for the TGF-*β*1 and collagen I expression levels as the gambir doses increased). The mean discrepancy for the NF-*κ*B levels was negative, meaning that the fibropreventive gambir and green tea groups had a higher mean NF-*κ*B level compared to BG. The higher the gambir dose, the higher the NF-*κ*B level.

AF G262 had the largest discrepancy among the antifibrotic groups in terms of the number of inflammatory cells (0.63) and the TGF-*β*1 (3.80) and NF-*κ*B (0.48) levels, followed by AF G131, which also had the largest discrepancy among the antifibrotic groups in terms of NF-*κ*B (0.48), TIMP-1 (11.74), and collagen I (14.50) levels. Western blot examination showed that the fibropreventive and antifibrotic groups had a specific band size of p65, whereas no specific band binding existed in the green tea group.

**Figure 7:**
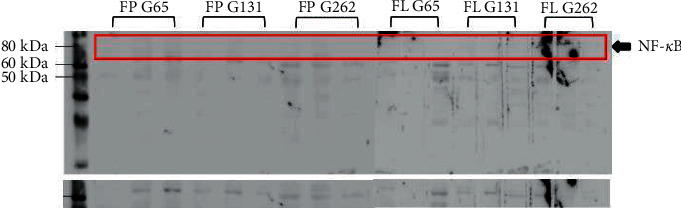
Western blot result on the gambir group. Western blot results showed that FP G65, FP G131, FP G262, FL G65, FL G131, and FL G262 had a specific band size of p65.

The Western blot assay revealed that the fibropreventive and antifibrotic groups of gambir were seen in a specific band of p65, as shown in Figure 7.

Meanwhile, the negative control group, the bleomycin group, and fibropreventive and antifibrotic groups of green tea extract did not show a specific band.

### 3.6. Gambir Is an Effective Antifibrotic Treatment

The results of our study demonstrated that gambir decreased the number of inflammatory cells, as well as the levels of TGF-*β*1, NF-*κ*B, TIMP-1, and collagen I expression. However, the increase in the dose of gambir was not in line with the decrease in the TGF-*β*1 and TIMP-1 expression levels. On the contrary, a lower dose of gambir produced a decrease in TGF-*β*1 levels and better TIMP-1 expression. The antifibrotic groups showed an increase in the gambir dose in line with a decrease in the TGF-*β*1 and NF-*κ*B levels, and lower TIMP-1 expression levels. This was supported by macroscopic and microscopic images that revealed less lung tissue damage and a less degree of fibrosis based on the modified Aschroft score, which was better than that for BG and other gambir groups.

## 4. Discussion

### 4.1. Green Tea and Gambir Administration Affect Pulmonary Tissue Damage

There are no studies examining the ability of gambir to reduce or prevent pulmonary fibrosis in humans and animals. Gambir is known to have a strong antioxidant effect because it contains abundant catechin [6–8]. Melia et al. revealed that besides its high antioxidant activity, gambir also exhibits antimicrobial activity [9]. Idiopathic pulmonary fibrosis (IPF), in which oxidative stress is the main pathway, is a fatal lung disease marked by chronic and progressive fibrosis that inhibits the functioning of the lungs [10]. According to Tsai et al. [11], the antifibrosis, anti-inflammatory, and antioxidative effects of catechin have been reported. Several in vitro and in vivo studies have shown that catechin can attenuate fibroblast proliferation, enhance antioxidant protection, and inhibit inflammation.

In this study, the administration of 262 mg/kg of bodyweight and 65 mg/kg of bodyweight of gambir to the antifibrotic groups and 65 mg/kg of bodyweight of gambir to the fibropreventive groups reduced macroscopic damage of pulmonary tissues compared to BG. This study showed that the administration of 131 mg/kg of bodyweight of gambir to the fibropreventive and antifibrotic groups resulted in extensive pulmonary tissue damage, characterized by pulmonary edema, lumps, bleeding, and a bluish-red tone. This may have been caused by the inhibition of the drug side-effect elimination system and the toxic effect of gambir at a certain dose on the lungs of animals.

### 4.2. The Administration of Green Tea Reduced the Inflammatory Cell Count

This study showed that the administration of green tea could reduce the number of inflammatory cells caused by intratracheal bleomycin. Fibrotic pulmonary tissue first undergoes cellular inflammation and histologically exhibits an accumulation of inflammatory cells, such as lymphocytes, macrophages, plasma cells, eosinophils, and neutrophils. Inflammatory response is one of the paths that leads to pulmonary fibrosis [12]. Dona et al. [13] stated that green tea strongly inhibits in vitro and in vivo neutrophil activities and, as an orally available medicine, may be effective in treating and preventing fibrosis.

Furthermore, the administration of green tea to the antifibrotic and fibropreventive groups also reduced the levels of TGF-*β*. TGF-*β* induces myofibroblast proliferation and extracellular matrix protein synthesis, suppresses ECM degradation by increasing antiproteinase or reducing metalloprotease which cause fibrosis. TGF-*β*1 also stimulates ROS production which leads to oxidative stress by reducing the antioxidant enzymes in the lungs and hepatocytes, such as catalase, GSH, and SOD. This can inhibit fibrogenic responses due to oxidative stress [14]. The administration of bleomycin to rats increased cytokine and chemokine levels on days 7 and 14 compared to the control group [15]. This study concluded that bleomycin administered intratracheally and fibropreventive green tea could reduce TGF-*β*1 levels. Tabuchi et al. [16] proposed that green tea could suppress TGF-*β*1 by interacting with type 2 TGF-*β* receptors.

NF-*κ*B is a type of regulator protein for the immune system and inflammatory responses [17]. Damaged pulmonary tissues activate NF-*κ*B through phosphorylation which mediated by I*κ*B kinase (IKK); degradation of inhibitory molecules, such as I*κ*B*α*; and phosphorylation of the NF-*κ*B p65 subunit. The activated NF-*κ*B then binds to the specific DNA region to regulate the expression of several inflammatory gene. In addition, NF-*κ*B regulates three main functions: inflammatory T cells activation, survivability, and the inflammatory responses. Activation of NF-*κ*B is responsible for profibrogenic signaling pathways. Based on the results of this study, bleomycin administration leads to a significant increase in NF-*κ*B levels, triggering inflammatory cytokines, such as TNF-*α* and IL-1*β*, and chemokines, such as MCP-1 (monocytes chemoattractant protein-1). S. Beigh et al. [18] found that TNF-*α* and transcription factor NF-*κ*B levels increased in fibrotic pulmonary tissue, induced by intratracheal 5 mg/kg bleomycin. Moreover, they stated that suppressing the NF-*κ*B signaling pathway could prevent the myofibroblast differentiation and fibrosis caused by bleomycin, followed by a reduction in *β*-catechin expression. Green tea, which has an antioxidant effect, inhibiting the activation of NF-*κ*B by suppressing collagen formation and TIMP-1 *through in vivo antifibrotic and fibropreventive mechanisms in the liver* [4, 19].

### 4.3. Administration of 262 mg/kg of Bodyweight of Gambir Reduced the Inflammatory Cell Count

The inflammatory cell count results of the BAL assessment showed that AF G262 had the lowest count, whereas BG had the highest. Tsai et al. [11] reported that several in vitro and in vivo studies indicated various effects of catechin on fibroblasts, such as suppressing cell proliferation, enhancing antioxidant protection, and inhibiting inflammation.

The TGF-*β*1 level were lowest in AF G262, followed by NG, and the highest in BG. Lago et al. [20] stated that flavonoid-type antifibrotic therapy could reduce TGF-*β*1 levels in the lungs of rats with fibrosis. You et al. [21] stated that epigallocatechin gallate (EGCG) could inhibit the proliferation of myofibroblasts by suppressing the secretion of TGF-*β*1.

The lowest NF-*κ*B level were found in AF G262, while the highest was in FP G131, followed by FP G262. The statistical test results did not reveal significant differences in the NF-*κ*B levels (*p*=0.089; *p* > 0.05). Gambir has proven to have an antioxidant effect via various mechanisms, including the inhibition of NF-*κ*B activation. Gambir acts as an antifibrotic agent, not only by inhibiting NF-*κ*B activation but also by its role as an anti-inflammatory and an antioxidant agent. Gambir may suppress the proinflammatory pathways related to most chronic diseases and inhibit the production of TNF and cell signaling mediated by TNF in various types of cells. Gambir has an antioxidant effect that can inhibit NF-*κ*B activation by suppressing the formation of collagen and TIMP-1 through in vivo antifibrotic and fibropreventive mechanisms in the liver [4, 5].

Paola [22] revealed that catechin reduced the exudate level of TNF-alpha, the active cytokines, and several signaling pathways, leading to the activation of the NF-*κ*B transcription factor. Polyphenol inhibited the induction of tumor necrosis factor-alpha in macrophages by suppressing the activation of NF-*κ*B.

### 4.4. Histopathological Imaging of Pulmonary Tissues Using Hematoxylin-Eosin and Masson's Trichome Staining Based on a Modified Ashcroft Score

From histopathological results based on the modified Ashcroft score, the green tea and gambir groups showed improvement, especially in AF G262, which exhibited a stage 3 modified Ashcroft score. The administration of preventive gambir in 131 mg/kg of bodyweight and 262 mg/kg of bodyweight doses produced histopathological images based on a stage 6 modified Ashcroft score, which tends to be more severe than other groups, although better than in BG. This indicated that the administration of preventive gambir and an increased dosage did not improve fibrosis in a dose-dependent manner. Other studies stated that the administration of bleomycin could increase the expression of inflammatory cytokines, followed by an increase in profibrotic mediators, such as TGF-*β*1, lysophosphatidic acid (LPA), and matrix deposition. The matrix metalloprotease (MMP) gene includes MMP12, MMP19, and lysyl oxidase-like (LOXL) [23]. Gambir and green tea are believed to suppress the expression of inflammatory cytokines and profibrotic mediators, such as TIMP-1 and collagen I. This was proved by Sri Ningsih in their study on the role of gambir extract in suppressing the development of liver fibrosis [19]. Seger et al. [24] who examined histopathological images of fibrotic lungs, showed an accumulation of fibrotic tissue and collagen deposition in the alveolar septum using specific Masson's trichome staining.

### 4.5. Administration of Bleomycin Increased TIMP-1 and Collagen I Expression Percentages

The lowest TIMP-1 expression was in NG, whereas the highest was in BG. TIMP-1 is believed to have an antiapoptotic effect on activated pulmonary epithelial cells. Hemmann [25] believes that MMP and its physiological inhibitor, TIMP-1, play an important role in the development of fibrotic tissue. Zhen et al. [26] revealed that during fibrosis progression, fibroblasts showed increased proliferation and migration, increased matrix protein expression, and production of MMP and TIMP, causing interstitial collagen or extracellular matrix substitution.

The lowest collagen I expression was in NG, whereas the highest was in BG. Green tea is believed to inhibit the formation of collagen I by increasing the production of MMP and triggering several types of cells, such as macrophages, to increase collagen I degradation. Salem et al. [27] claimed that a green tea group showed thin interalveolar septa, decreased cellular infiltration, red blood cells, and accumulation of collagen fibers. Immunohistochemistry staining was represented by a brown color.

### 4.6. Administration of 262 mg/kg of Bodyweight Gambir to the Antifibrotic Groups Decreased the TIMP-1 Expression Percentage, and Administration of 131 mg/kg of Bodyweight of Gambir to the Antifibrotic Groups Decreased the Collagen I Expression Percentage

The lowest TIMP-1 expression was in AF G262, whereas the highest was in BG. The PCR results in the study conducted by Dona et al. [13] showed that catechin decreased the collagen I and TIMP-1 transcription. Therefore, catechin is believed to be a potential therapy for liver fibrosis.

Based on microscopic images, TIMP-1, and collagen I expressions were marked by well-defined brown fibers along alveolar septa. There were various TIMP-1 and collagen I expressions in all groups, but the differences were not significant. Tissue inhibitors of metalloproteinase (TIMPs) are specific and endogenous inhibitor proteins of matrix metalloproteinases (MMPs). Increased TIMP levels result in ECM accumulation, which causes pulmonary fibrosis [28]. In this study, the mean percentages for TIMP-1 and collagen I expressions between groups indicated that AF G262 had a lower percentage than that of BG, although it was higher than that of NG. This was supported by Sri Ningsih, who revealed that the administration of gambir extract decreased TIMP-1 and collagen I expression, which then suppressed the development of liver fibrosis [19].

The lowest collagen I expression was in AF G131, and the highest was in BG. FP G131 had lower collagen I expression than the other fibropreventive groups and BG, although it was higher than in NG. AF G131 had lower collagen I expression than the other antifibrotic groups and control groups (NG and BG). This was caused by the dose-dependent antioxidant activity of gambir, with a lower dose producing a suboptimal response and a higher dose potentially being toxic, which is consistent with Tsai et al. [11]. Nakamuta et al. [29] concluded that the dose of catechin affected the production of collagen and MMP activity.

Antifibrotic doses of 131 mg/kg of bodyweight gambir are believed to inhibit the formation of collagen I by increasing the production of MMP and triggering several types of cells, such as macrophages, thus increasing collagen I degradation. Chen et al. [30] stated that the calculation and identification of extracellular matrix and collagen I expression could be conducted by immunohistochemistry staining and image analysis techniques.

### 4.7. Interaction between Gambir and NF-*κ*B Using the Western Blot Method

**Figure 8:**
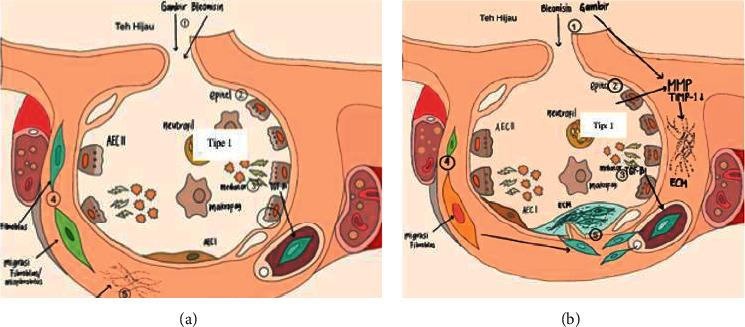
Mechanism of (a) fibropreventive and (b) antifibrotic of gambir. (a) (1) Gambir and green tea were given initially to suppress the production of ECM by interrupting the activation of type 1 pulmonary epithelial cells, suppressing the activities of ROS, mediators, and inflammatory cells, followed by the administration of bleomycin at the beginning of weeks 2 and 4. (2) The damage to type 1 pulmonary epithelial tissue is decreased. (3) The activities of ROS, inflammatory cells (including neutrophils, macrophages, and lymphocytes), and the activities of the inflammatory mediators TGF-*β*1, NF-*κ*B, and TIMP-1/collagen decreased. (4) The activation of fibroblasts and myofibroblast proliferation decreased. (5) The deposition of the extracellular matrix (especially in FP GT) decreased. b) (2) Bleomycin was given first to initiate fibrosis, followed by the administration of gambir and green tea at the beginning of week 3 to lyse ECM and suppression of the activation of type 1 epithelial cells. (2) Type 2 pulmonary epithelial damage improved. (3) The activities of ROS, inflammatory cells (including neutrophils, macrophages, and lymphocytes), and inflammatory mediator TGF-*β*1, NF-*κ*B, and TIMP-1/collagen expression levels were suppressed. (4) The activation level of fibroblasts and myofibroblasts decreased. (5) Lysis of the extracellular matrix occurred (especially in AF G262).

Western blot assays revealed that FP G65, FP G131, FP G262, AF G65, AF G131, and AF G262 had a specific p65 NF-*κ*B dimer-size band. This indicated that the bonding interaction of gambir with NF-*κ*B could prevent the translocation to the nucleus, thus no initiation of the inflammatory/fibrogenesis process. Bitzer et al. [31] confirmed that the protective effect of an antifibrosis agent was correlated to a high level of Smad7 and inactivation of the NF-*κ*B and TGF-*β*1 signaling pathways, showed by performing Western blot analysis on I*κβα* and NF-*κ*Bp65 antibodies. The fibropreventive and antifibrotic mechanisms of green tea and gambir identified in this study are shown in Figure 8.

Many studies have demonstrated the effectiveness of green tea in inhibiting pulmonary fibrosis. The fibropreventive effect of green tea proved to be better than gambir, a result supported by El-Safti et al. [32]. Due to different methods of administration, pulmonary fibrosis improvement might occur through natural recovery after arresting bleomycin induction in the moderate stage of fibrosis. Increasing the dose of gambir did not have the expected fibropreventive effect related to the dose-dependent antioxidant activity of gambir, as stated by Tsai et al. [11].

The administration of preventive gambir in 131 mg/kg of bodyweight and 262 mg/kg of bodyweight doses did not result in low-stage histopathological images based on a stage 6 modified Ashcroft score, which tend to be worse than in the other groups, although better than in BG. This showed that the administration of preventive gambir and an increase in the dose did not improve fibrosis in a dose-dependent manner. AF G262 showed a reduced (stage 3) modified Ashcroft score/histopathological image and decreased inflammatory cell counts and levels of TGF-*β*1, TIMP-1, and NF-*κ*B, which prompted the formation of fibrosis; therefore, the administration of 262 mg/kg of bodyweight of gambir could improve fibrosis/lyse ECM in rat lungs.

In summary, AF G262 had the largest discrepancy among the antifibrotic groups in terms of the number of inflammatory cells, the TGF-*β*1 and NF-*κ*B levels. Western blot also showed that the fibropreventive and antifibrotic groups had a specific band size of p65, whereas no specific band binding existed in the green tea group. The results of our study demonstrated that gambir decreased the number of inflammatory cells, as well as the levels of TGF-*β*1, NF-*κ*B, TIMP-1, and collagen I expression. Further studies are needed to consider MMP, ROS, and fibroblast counts after the administration of 262 mg/kg of bodyweight of gambir for 7 weeks in animal models. Further studies are also needed to determine the safety of gambir for humans at a dose equivalent to 262 mg/kg of bodyweight for seven weeks.

## Figures and Tables

**Table 1:** Characteristics and macroscopic imaging of rat lungs.

Group *n* = 3	Bodyweight, mean (g)	Lung weight, mean (g)	Relative organ weight	Macroscopic image
NG	218	3.7	0.016	No damage to the lungs.
BG	232	4.3	0.018	Damage to the lungs, marked by bleeding, edema, and lumps.
FP GT	246	4	0.016	Little damage to the lungs.
AF GT	235	3.3	0.014	Little damage to the lungs.
FP G65	212	3	0.014	Little damage to the lungs, with several parts seeming normal.
FP G131	243	3.9	0.016	Extensive damage to the lungs, marked by edema, lumps, bleeding, and a bluish-red tone.
FP G262	210	2.8	0.013	Damage to the lungs, bleeding, and lumps, but not as severe as for FP G131.
AF G65	221	4	0.018	Little damage to the lungs, and several parts seemed normal.
AF G131	240	4.1	0.017	Extensive damage to the lungs, marked by edema, lumps, bleeding, and a bluish-red tone.
AF G262	229	3	0.013	Little damage to the lungs, and several parts seemed normal.

## Data Availability

Data are available upon request.
